# Chitosan-Based Hydrogel Beads: Developments, Applications, and Challenges

**DOI:** 10.3390/polym17070920

**Published:** 2025-03-28

**Authors:** Ziao Li, Ruoran Qin, Jiayi Xue, Congyu Lin, Longwei Jiang

**Affiliations:** 1 School of Food and Nutrition, Anhui Agricultural University, Hefei 230036, China; liziao0502@163.com (Z.L.); 18355935022@163.com (R.Q.); xuejiayi0205@163.com (J.X.); 2 School of Food Science and Technology, Jiangnan University, Wuxi 214122, China; lincongyu@jiangnan.edu.cn

**Keywords:** chitosan-based hydrogel beads, preparation method, food preservation, drug transportation, environmental protection

## Abstract

Currently, as research on hydrogel beads intensifies, the application scope of chitosan-based hydrogel beads is increasingly expanding. Owing to their unique three-dimensional network structure, chitosan-based hydrogel beads are frequently utilized for encapsulating bioactive substances and adsorbing impurities. The primary material used in the preparation of chitosan-based hydrogel beads is chitosan, which is uniquely a natural polysaccharide possessing a positive charge. Derived from a diverse array of sources, chitosan is non-toxic, exhibits excellent biocompatibility, and possesses certain antibacterial properties. Because of these remarkable attributes, it has found widespread application in tissue engineering, the formulation of drug carriers, and the adsorption of heavy metals and dyes in wastewater. The preparation method for chitosan-based hydrogel beads largely mirrors that of other hydrogel beads. According to existing research, numerous methods exist for crafting hydrogel beads with diverse properties. This paper reviews the preparation methods of chitosan-based hydrogel beads, encompassing both physical and chemical crosslinking techniques. The physical crosslinking method leverages electrostatic interactions between materials to form hydrogel beads, whereas the chemical crosslinking method involves the use of chemical crosslinking agents to facilitate the formation of hydrogel beads through material-based chemical reactions. Given that chitosan carries a positive charge and other polysaccharide materials possess a negative charge, the combination of these materials can yield hydrogel beads with a dense structure, effectively encapsulating bioactive substances. This dense internal structure offers superior protection for the encapsulated bioactive substances. Chitosan-based hydrogel beads typically feature large pore sizes, providing numerous adsorption sites, which makes them well suited for wastewater treatment. Additionally, this paper examines the recent applications of chitosan-based hydrogel beads in food preservation, medicine, and environmental protection. Starting with the materials and methods for preparing chitosan-based hydrogel beads, this paper delves into their applications in food preservation, biomedicine, and environmental protection, offering insights for future developments and applications of chitosan-based hydrogel beads and fostering further innovation and advancement in this field.

## 1. Introduction

**Figure 1:**
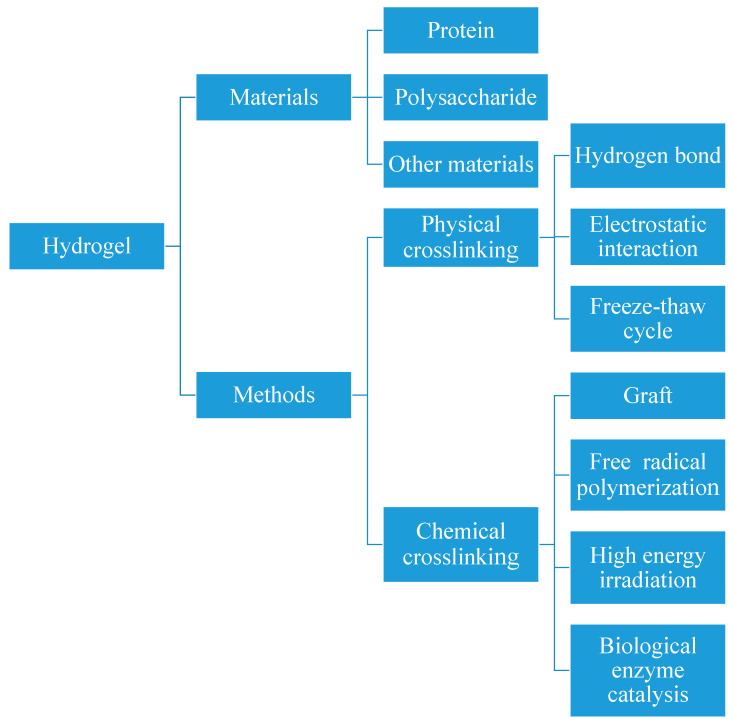
The preparation material and method of hydrogel.

Hydrogel is a three-dimensional network structure formed by polymer polymerization or crosslinking [1]. Benefiting from their unique internal architecture, hydrogels exhibit limited swelling in aqueous environments while maintaining structural integrity. These materials possess numerous advantageous properties, including excellent biocompatibility, biodegradability, and potential utility as drug delivery carriers. Therefore, the application of hydrogels can be seen in the fields of food, environmental protection and medicine. Hydrogels can be fabricated using various materials and methods, as illustrated in Figure 1. The two most common materials used to produce hydrogels are polysaccharides and proteins, but there are other materials that can also be used to produce hydrogels. In this paper, the preparation methods of hydrogels and hydrogel beads prepared from polysaccharides (such as chitosan) and some proteins were discussed, and the research progress of chitosan-based hydrogel beads in recent years was described. Regarding production methodologies, hydrogel preparation can be categorized into two primary approaches: physical crosslinking and chemical crosslinking. The physical crosslinking method primarily relies on hydrogen bonding and electrostatic interactions between materials to form polymeric networks, thereby creating hydrogels [2]. A notable example involves the utilization of electrostatic interactions between chitosan and sodium alginate, two polysaccharides, combined with hydrogen bond formation to create a polyelectrolyte complex. This process has been successfully employed to produce hydrogel beads encapsulating *Perilla frutescens* L. essential oil. The experiments showed that the compact structure of hydrogel beads could protect *Perilla frutescens* L. essential oil well, and the encapsulation efficiency and loading rate of *Perilla frutescens* L. essential oil were 61.29 and 41.11%, respectively [3]. Furthermore, research has demonstrated the feasibility of constructing hydrogels through the blending of polysaccharides and proteins. For instance, a curcumin-encapsulating hydrogel has been developed through electrostatic interactions between gum Arabic (a polysaccharide) and gelatin (a protein) [4]. The results showed that the prepared hydrogel beads could improve the antioxidant capacity of curcumin, and the scavenging capacity of DPPH and ABTS free radicals was 95.59% and 87.65%, respectively. At the same time, hydrogel beads can also effectively delay the action time of curcumin. Beyond electrostatic interactions and hydrogen bonding, physical crosslinking can also be achieved through freeze–thaw cycles. A practical application of this method involves using polyvinyl alcohol and starch as base materials, incorporating black wolfberry anthocyanins into the solution. Through repeated freeze–thaw cycles, researchers have developed a hydrogel capable of monitoring mutton freshness. Additionally, owing to the inherent antibacterial properties of anthocyanins, the resulting hydrogel effectively extends the shelf life of mutton [5].

In addition to physical crosslinking methods, chemical crosslinking represents another prevalent approach for hydrogel synthesis. The primary chemical methods employed in hydrogel preparation include grafting copolymerization, free radical polymerization, high-energy irradiation crosslinking, and enzyme-catalyzed crosslinking. Grafting copolymerization involves the polymerization of monomers onto the backbone of prefabricated polymers. For instance, researchers have developed a hybrid polymer by utilizing N-(2-hydroxypropyl) methacrylamide, which was subsequently assembled with peptide grafts of varying lengths [6]. The results show that the three-dimensional structure formation of hydrogel beads requires a graft containing at least four heptapeptides. At the same time, the increase in the number of heptapeptides in the graft from four to five may lead to increased participation of peptide self-assembly in the gelation process. Free radical polymerization is extensively utilized in hydrogel fabrication. This process is initiated by free radicals in the presence of crosslinking agents, facilitating chain growth and ultimately leading to the polymerization of numerous monomeric units into three-dimensional network structures. A representative example of this method is the synthesis of N-isopropylacrylamide (NIPA)-based gels. Through free radical polymerization, thermally responsive hyaluronic acid gels have been successfully developed using NIPA as the primary monomer [7]. The principle underlying high-energy irradiation for hydrogel preparation involves the derivation of water-soluble polymers through vinyl groups under gamma or electron beam radiation. This process generates free radicals on polymer chains via homolytic cleavage, which subsequently recombine across different chemical chains to form covalent bonds, ultimately yielding crosslinked structures. For instance, researchers have successfully produced highly absorbent hydrogels by irradiating carboxymethyl cellulose derivatives with gamma rays in the presence of crosslinkers [8]. The results showed that the water absorption capacity of hydrogel beads prepared by the enzymic crosslinking method with gelatin and hyaluronic acid as raw materials decreased with the increase in gelatin content. Enzymatic crosslinking offers distinct advantages in hydrogel synthesis, characterized by mild reaction conditions and the utilization of predominantly non-toxic crosslinkers, resulting in hydrogels with excellent biocompatibility. A notable example is the development of gelatin/hyaluronic acid hydrogels with interpenetrating network structures through dual-enzyme crosslinking using transglutaminase. These hydrogels demonstrate significant potential and promising applications in tissue engineering, wound healing, and drug delivery systems [9]. Beyond single-method approaches, researchers frequently employ combined techniques to fabricate hydrogels with enhanced properties. Hydrogel beads, a specific form of hydrogel, can be produced through these methods by either injecting hydrogel solutions into fixed molds or utilizing syringe-based techniques. The choice of preparation method significantly influences the mechanical strength, structural uniformity, and overall characteristics of the resulting hydrogel beads. For example, physical crosslinking methods that exploit electrostatic interactions between chitosan and other substances can substantially affect both the morphology and mechanical properties of chitosan-based hydrogel beads. Conversely, chemical crosslinking enhances the internal crosslinking density of hydrogel beads, thereby improving their overall performance. In this context, hydrogel beads refer to macroscopic spherical structures typically measuring several millimeters in diameter, distinct from microscopic particles. These spherical hydrogel structures are fabricated either by dripping gel solutions into crosslinkers using syringes or by employing fixed molds for shape formation.

**Figure 2:**
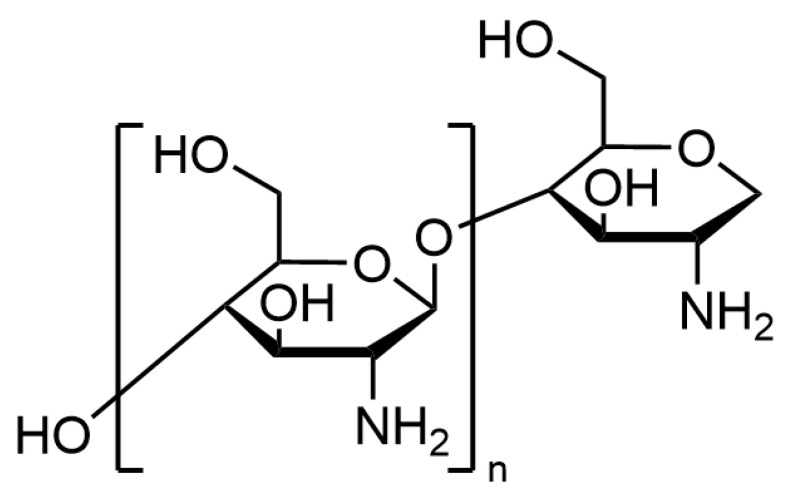
Chemical structure of CS.

Hydrogel beads play a significant role in the food, medical, and environmental fields due to their unique spherical shape and high specific surface area. The bead-like structure of hydrogels enhances their usability. Additionally, the small size of hydrogel beads allows them to be incorporated into food matrices without compromising the sensory quality of the food. In contemporary food research, hydrogel beads have garnered considerable attention from researchers due to their aesthetically pleasing spherical appearance. Typically, the diameter of these beads is on the millimeter scale. For instance, some researchers have prepared hydrogel beads with diameters ranging from 3.79 to 4.23 mm [10], while others have produced beads with diameters between 2.6 and 5.6 mm [11]. Hydrogel beads can be fabricated from a variety of raw materials, with chitosan (CS) being the most commonly used. CS is the second-most abundant biopolymer on Earth, following cellulose. It is extracted from the shells of shrimp and other crustaceans. As illustrated in Figure 2, the chemical structure of CS is clearly depicted. CS is a copolymer formed of repeated units of 2-amino-2-deoxy-D-glucopyranose units, and residual 2-acetamido-2-deoxy-D-glucopyranose units [12]. The properties of CS are affected by its molecular weight (usually 1–5 × 10^5^ g/mol), purity, and degree of deacetylation. The acetylation degree refers to the ratio of N-acetyl-d-glucosamine units in CS to the total number of units, and the typical acetylation degree of CS is less than 50% [13]. CS exhibits excellent biodegradability, non-toxicity, biocompatibility, antibacterial properties, and heavy ion adsorption capabilities. It is also widely available and cost-effective [10]. Therefore, CS is the primary material of choice for fabricating hydrogel beads. Compared to other naturally derived polymers, although CS is a macromolecular substance, it is unique as the only positively charged polysaccharide in nature. Researchers have conducted extensive experimental studies on CS. However, despite its numerous advantages, hydrogel beads prepared using CS still face several limitations, such as inconsistent yield, non-uniform sizes, lack of process repeatability, and poor mechanical strength and chemical resistance [14]. To address these issues, researchers often incorporate new materials in specific proportions with CS to produce CS-based hydrogel beads with enhanced performance. There are various methods for preparing CS-based hydrogel beads, including techniques that utilize the inherent properties of CS and chemical methods that employ crosslinking agents to facilitate hydrogel bead formation. With the continuous advancement of science and technology, it is anticipated that more innovative methods for preparing CS-based hydrogel beads will emerge in the future.

CS is easily soluble in acid and insoluble in alkali [15], and leveraging this property, researchers can prepare CS-based hydrogel beads by dropping a CS solution into a NaOH solution using a syringe. For instance, CS is first dissolved in a 2% (*v*/*v*) acetic acid solution, and the resulting solution is then dropped into a NaOH solution via a syringe. After 10 min, the hydrogel beads are removed and rinsed with distilled water until neutral, yielding CS-based hydrogel beads loaded with proanthocyanidins [11]. In addition, CS is a positively charged cationic polysaccharide, and it can form electrostatic interactions and hydrogen bonds with negatively charged materials. By exploiting the opposite charges of CS and sodium alginate, a complex can be formed. Hydrogel beads encapsulating nifedipine can be prepared using a calcium chloride solution as a crosslinking agent [16]. The dense internal structure of these hydrogel beads ensures effective protection of the encapsulated drug. The materials used for preparing hydrogel beads are not limited to CS; sodium alginate is also widely utilized for this purpose. In terms of encapsulation efficiency, comparative studies have shown that CS-based hydrogel beads exhibit comparable performance to other hydrogel systems while offering cost advantages. For instance, CS-based hydrogel beads demonstrate an encapsulation rate of 61.29% for *Perilla frutescens* L. essential oil [3], which is higher than the 50.59% encapsulation rate achieved by sodium alginate hydrogel beads for curcumin [4]. Regarding degradation and pH sensitivity, both materials possess favorable properties. CS is a non-toxic carbohydrate with excellent biocompatibility and biodegradability [17], and sodium alginate shares similar characteristics. Although both materials are pH-sensitive, sodium alginate hydrogel beads formed by calcium ion crosslinking exhibit limited swelling at pH 7.4, which can affect their performance as bioactive ingredient delivery systems [17]. In contrast, CS-based hydrogel beads demonstrate a more favorable release profile: they release bioactive ingredients slowly in acidic gastric fluid and accelerate release in alkaline intestinal fluid [18]. Consequently, CS-based hydrogel beads offer broader application potential compared to sodium alginate hydrogel beads. Additionally, the preparation method for CS-based hydrogel beads is relatively simpler, further enhancing their practical utility.

It is evident that CS-based hydrogel beads not only offer diverse preparation methods but also find applications across various fields. The compact structure of CS-based hydrogel beads makes them promising candidates for drug delivery systems and suitable for encapsulating bioactive substances to enhance food preservation. Furthermore, CS-based hydrogel beads contain numerous adsorption sites, enabling them to effectively adsorb heavy metals and pharmaceutical compounds from wastewater. This paper primarily explores the applications of CS-based hydrogel beads in the fields of food, biomedicine, and environmental science, providing insights and a theoretical foundation for future research on CS-based hydrogel beads.

## 2. Research Progress of CS-Based Hydrogel Beads

### 2.1. Research Progress in Preparation of CS-Based Hydrogel Beads

In recent years, CS-based hydrogel beads have been developed through various methods and applied in diverse fields. In 2018, researchers explored the use of CS to prepare hydrogel beads for transporting trace elements, demonstrating their potential as human nutritional supplements. For instance, a chemical crosslinking method was employed to produce hydrogel beads containing iron ions. This was achieved by extruding a CS solution into an iron ion solution, creating a fortifying agent capable of supplementing iron in the human body [19]. Additionally, a CS-positive charge enables it to interact electrostatically with the negatively charged lipids and proteins in bacterial cell walls. This interaction disrupts bacterial biofilms, leading to effective bactericidal activity [20]. Researchers have also utilized the ion gelation method to prepare CS-based hydrogel beads, which have shown significant antibacterial effects against *Escherichia coli* and *Staphylococcus aureus*. These findings suggest their potential application as advanced wound dressings [21].

In 2019–2020, researchers focused increasingly on the application of CS-based hydrogel beads for the delivery of bioactive ingredients and drugs. For instance, some researchers developed CS-based hydrogel beads containing flaxseed oil and quercetin using a chemical crosslinking method. The process involved dropping a CS solution into a sodium phosphate solution via a syringe. After crosslinking, the resulting hydrogel beads were thoroughly washed and dried. The findings revealed that these CS hydrogel beads not only enhanced the stability of flaxseed oil and quercetin but also prolonged their retention time in the gastrointestinal tract, thereby improving their oral bioavailability [22]. In addition to encapsulating bioactive compounds, researchers also employed direct precipitation and physical methods to prepare CS-based hydrogel beads loaded with diclofenac sodium. In this approach, a CS solution was dropped into a NaOH solution to form hydrogel beads, which were then immersed in a sodium alginate solution. The two components were combined through electrostatic interactions, resulting in a compact structure that significantly delayed drug release and extended its therapeutic duration [23].

In 2021–2023, CS-based hydrogel beads were further investigated as drug carriers. For example, studies utilized chemical crosslinking methods to prepare CS-based hydrogel beads loaded with resveratrol [24]. Owing to CS-abundant amino and hydroxyl functional groups, which provide numerous adsorption sites [18], researchers also developed CS-based hydrogel beads for diverse applications. These included hydrogel beads prepared via irradiation and grafting for dye adsorption [25] and those synthesized through ion crosslinking for heavy metal adsorption [26]. Furthermore, CS-based hydrogel beads were employed to immobilize glucase as a micro-catalyst for glucose production [27] and to fix protease, enabling efficient enzyme reuse [28]. In the most recent years (2024–2025), there has been a growing emphasis on the preparation of CS-based hydrogel beads using physical methods. Leveraging CS insolubility in alkaline solutions, researchers dropped CS solutions into lye through syringes to form hardened hydrogel beads. For example, physical methods were used to prepare CS-based hydrogel beads capable of adsorbing hexavalent chromium ions [29] and encapsulating proanthocyanidins for sustained drug delivery [11].

### 2.2. Advantages and Disadvantages of Different Preparation Methods

Generally speaking, CS-based hydrogel beads prepared by chemical methods exhibit excellent mechanical properties. However, most chemical crosslinking agents are toxic, and it is challenging to ensure their complete removal during the preparation process. In contrast, the physical preparation of CS-based hydrogel beads relies on hydrogen bonding, electrostatic interactions between materials, or the inherent properties of CS. While the resulting hydrogel beads are safe and non-toxic, they often suffer from poor mechanical properties and typically require combination with other materials to enhance their performance. Therefore, identifying suitable materials to complement CS and developing physical methods for preparing CS-based hydrogel beads represent key future research directions in this field.

## 3. Application of CS-Based Hydrogel Beads

### 3.1. Application of CS-Based Hydrogel Beads in Food

**Figure 3:**
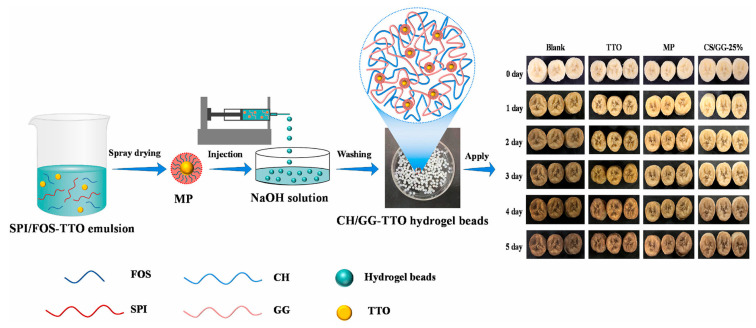
The preservation of bananas by hydrogel beads [31].

Owing to their small volume and high specific surface area, hydrogel beads can be easily incorporated into food substrates without compromising the sensory and nutritional properties of the food [30]. The compact internal network structure of these beads enables effective protection of encapsulated bioactive substances and allows for controlled release of these compounds. Consequently, researchers frequently utilize hydrogel beads to encapsulate various bioactive substances, including natural plant essential oils and other active compounds with antibacterial and antioxidant properties, thereby extending the shelf life of food products. As illustrated in Figure 3, based on our previous research, we have successfully prepared hydrogel beads using CS and gellan gum. The hydrogel beads containing tea tree oil (TTO) were put into a non-woven bag, and the non-woven bag was placed in the box with bananas to explore the fresh-keeping effect of the hydrogel beads on bananas. The results showed that the hydrogel beads containing TTO could prolong the shelf life of bananas well, and the freshness indexes such as the hardness of bananas were maintained well [31]. Hydrogel beads can also encapsulate color-responsive chemicals and natural products (such as polydiacetylene and anthocyanins). Hydrogel beads containing the above substances can be used as an indicator of meat spoilage, and since the extraction cost of CS is low, it is a good strategy to use CS-based hydrogel beads to monitor the change of meat freshness. Meat in the storage process will produce biological amines and other alkaline substances, and polydiacetylene is sensitive to acid–base and temperature, easy to change color. Polydiacetylene was added to the hydrogel to produce hydrogel beads that can monitor meat freshness [32]. Because the dense structure inside the hydrogel beads can protect these color-responsive substances against the invasion of the external environment, it is suitable as an indicator of meat freshness. On the basis of safety and environmental protection, consumers prefer to use substances that exist in nature. Nature provides numerous color-responsive substances, among which anthocyanins are particularly noteworthy. Anthocyanins, a class of flavonoid compounds characterized by a 2-phenylbenzopyranoid cation as the parent nucleus conjugated with one or more sugar groups, represent water-soluble pigments that are widely distributed in various plant species [33]. These compounds exhibit remarkable sensitivity to pH changes, demonstrating distinct color variations under different acid–base conditions [34]. During storage, fish undergo oxidative decomposition and microbial growth, leading to the production of volatile basic nitrogen compounds. When hydrogel beads encapsulating anthocyanins were co-stored with fish, researchers observed that the bead color changed in response to increasing fish spoilage and rising volatile basic nitrogen levels [35]. Similarly, pork also generates volatile basic nitrogen during storage. Researchers have successfully employed anthocyanin-containing hydrogel beads to monitor pork freshness, demonstrating significant color changes in the beads corresponding to extended storage periods [36]. Furthermore, CS-based hydrogel beads have been developed to encapsulate various bioactive substances for applications in both food preservation and freshness monitoring. In Section 3.1.1 and Section 3.1.2, the application of CS-based hydrogel beads in fruit preservation and meat preservation will be described in detail.

#### 3.1.1. Application of CS-Based Hydrogel Beads in Fruit Preservation

CS-based hydrogel beads are widely utilized to extend the shelf life of fruits, often encapsulating antioxidant compounds or bioactive substances with antibacterial and antioxidant properties. The compact structure of CS-based hydrogel beads serves to enhance their protective function and effectively regulate the release of internal active substances. For instance, research has demonstrated the combination of hydrogel beads with tea tree oil (TTO) for fruit preservation. TTO, a natural plant essential oil, exhibits antifungal, antiviral, and antiparasitic properties [37]. Due to these exceptional characteristics, TTO finds extensive applications in both cosmetic and food industries. However, similar to other natural plant essential oils, TTO is volatile, environmentally sensitive, inherently unstable, and not suitable for direct application [38]. The encapsulation of TTO within CS-based hydrogel beads leverages the dense network structure of the beads to shield the oil from external oxygen, thereby enhancing its stability. TTO has proven effective in fruit preservation; for example, in pineapple preservation, it can induce resistance responses, reduce water loss, and maintain fruit firmness [39]. Additionally, TTO possesses antimicrobial properties that inhibit microbial growth and proliferation. Beyond TTO encapsulation, CS-based hydrogels can also encapsulate various other essential oils, such as garlic essential oil, which effectively inhibits microorganisms like *Escherichia coli* and *Listeria*, serving as natural preservatives in food storage [40]. Utilizing CS and sodium alginate as shell materials, researchers have encapsulated microcapsule powder containing *Perilla frutescens* L. essential oil for strawberry preservation, demonstrating that hydrogel beads can significantly extend the shelf life while maintaining the sensory quality [41]. Furthermore, these hydrogels can encapsulate substances like resveratrol and polyphenols, which also exhibit antimicrobial and antioxidant capabilities, making them potential candidates for fruit preservation applications. Generally, the encapsulation efficiency of hydrogel beads varies with the quantity of encapsulated substance added [4]. When bioactive substances (such as essential oils and antioxidants) are encapsulated, the hydrogel beads’ dense structure controls their release, prolonging their efficacy. For example, researchers have prepared hydrogel beads containing coffee essential oil using pectin and CS through physical crosslinking, showing that these beads possess excellent digestive resistance and serve as effective carriers for essential oils [42]. The higher the encapsulation efficiency, and the slower the release rate of bioactive substances from hydrogel beads, the better the preservation effect on food, and the longer the preservation duration.

#### 3.1.2. Application of CS-Based Hydrogel Beads in Meat Preservation

During the storage of meat, significant microbial growth and oxidative deterioration lead to the production of biological amines, including volatile base nitrogen and other compounds. For instance, microorganisms such as Pseudomonas on the surface of fish and meat can degrade proteins, generating volatile amines like ammonia, histamine, putrescine, and cadaverine, which create an alkaline environment. Anthocyanins exhibit noticeable color changes in response to pH variations. Consequently, CS-based hydrogel beads containing anthocyanins can undergo color changes as meat spoilage progresses. Additionally, anthocyanins possess antibacterial and antioxidant properties, which can help extend the shelf life of meat. Hydrogel beads can encapsulate anthocyanins to monitor meat freshness. For example, anthocyanins incorporated into sodium alginate hydrogel beads have been used to monitor the freshness of chicken and shrimp. The results demonstrated that these hydrogel beads could significantly change color in response to freshness variations [43]. Similarly, researchers have mixed sodium alginate solutions with flavonoid solutions to prepare hydrogel beads that are highly sensitive to the freshness of salads and kale [44]. While numerous studies have explored the use of various materials to prepare hydrogel beads for meat preservation, research on CS-based hydrogel beads for this purpose remains limited. Therefore, future studies could focus on selecting materials sensitive to alkaline substances, such as volatile base nitrogen, as the core material, with CS-based hydrogel beads serving as the shell. This approach could yield hydrogel beads that not only extend the shelf life of meat but also monitor its freshness, representing a promising direction for future research.

### 3.2. Application of CS-Based Hydrogel Beads in Medicine

The amino protonating groups of CS exhibit the ability to bind with negatively charged components (lipids and proteins) of bacterial cells, leading to bacterial cell death [20]. Furthermore, CS demonstrates the capacity to interact with bacterial DNA, thereby interfering with mRNA transcription and subsequent protein synthesis. Therefore, CS in the medical field is usually made into a hydrogel as a wound dressing, which has a good effect in local hemostasis. For example, researchers have successfully developed spongy antibacterial materials with CS nanostructures by incorporating zinc oxide nanoparticles into CS solutions, which have been effectively utilized as local hemostatic agents [45]. The widespread investigation of CS in biomedical and pharmaceutical applications can be attributed to its remarkable properties, including non-toxicity, excellent biocompatibility, and biodegradability [46]. In a representative study, scientists enhanced hydrogel beads fabricated from CS and sodium alginate by incorporating longan seed extract. The experimental results demonstrated that the resulting hydrogel beads exhibited superior mechanical strength and controlled drug release characteristics, suggesting their promising potential as advanced wound dressing materials [47]. In addition to serving as wound dressings, CS-based hydrogel beads can also be utilized as drug delivery systems for targeted drug administration to various parts of the human body. Some studies have employed high-energy radiation, such as gamma rays, to fabricate hydrogel beads. These studies incorporate anticancer drugs into the hydrogel matrix, resulting in the development of highly porous, pH-responsive, and biocompatible CS-based hydrogel beads [25]. As a drug delivery system, CS-based hydrogel beads can not only protect drugs from the invasion of the external environment but also effectively control the release of drugs. In other studies, researchers have utilized CS as a raw material to prepare hydrogel beads for encapsulating proanthocyanidins, demonstrating effective protection of these compounds. The findings reveal that such hydrogel beads can effectively regulate the release rate of proanthocyanidins [11]. Similarly, in research focusing on drug encapsulation and delivery, attempts have been made to combine CS with carrageenan and sodium alginate to fabricate hydrogel beads capable of encapsulating caffeine. The results indicate that hydrogel beads composed of CS and sodium alginate can significantly prolong the release of caffeine [48]. Among various hydrogel beads employed as drug delivery systems, magnetic hydrogel beads have garnered significant attention due to their unique properties. These beads can be fabricated from diverse raw materials, including polysaccharides and proteins [49]. Magnetic hydrogel beads possess distinctive characteristics that distinguish them from conventional hydrogel beads, particularly their capability for targeted drug delivery and sustained release. Numerous studies have explored drug delivery using CS-based hydrogel beads. For instance, researchers have developed magnetic hydrogel beads with dual responsiveness (magnetic and pH) by combining carrageenan and CS with iron oxide nanoparticles [50]. Investigations on diclofenac sodium release have demonstrated the drug delivery potential of these hydrogel systems. Furthermore, researchers have successfully prepared magnetically responsive hydrogel beads through complexation of CS and pectin, using metamizole as a model drug [51]. The results indicate that these hydrogel beads can function as intelligent drug delivery systems with potential applications in localized cancer treatment. These studies collectively demonstrate that CS-based hydrogel beads not only serve as effective wound dressings but also exhibit remarkable potential as advanced drug delivery systems.

### 3.3. Application of CS-Based Hydrogel Beads in Environmental Treatment

With the increasing water consumption in cities, the volume of wastewater requiring treatment has also risen significantly. Wastewater typically contains various impurities, including heavy metal ions, dyes, and pharmaceutical residues. The adsorption of these contaminants using hydrogel beads has become a widely adopted treatment method. Numerous studies have been conducted on the application of CS-based hydrogel beads for the removal of impurities from wastewater.

#### 3.3.1. CS-Based Hydrogel Beads Adsorbed Heavy Metal Ions in Wastewater

**Figure 4:**
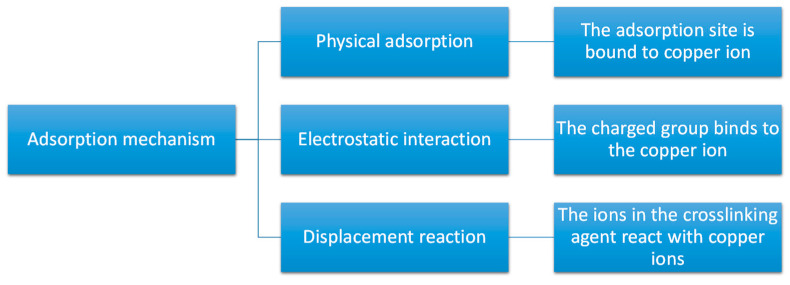
Mechanism of the adsorption of copper ions by hydrogel beads.

There have been numerous studies on the utilization of CS-based hydrogel beads for adsorbing heavy metal ions in wastewater. As illustrated in Figure 4, using the adsorption of copper ions as an example, the primary mechanisms of copper ion adsorption by hydrogel beads are as follows. Firstly, owing to the network structure of CS-based hydrogel beads, they contain a multitude of adsorption sites internally, which can bind with a significant number of copper ions, facilitating the physical adsorption of these ions. Secondly, copper ions can attach to certain charged groups (such as hydroxyl, carboxyl, and amino groups) within the CS-based hydrogel beads, leveraging the electrostatic interactions between them. Lastly, if a calcium chloride solution is employed as a crosslinking agent during the preparation of CS-based hydrogels, calcium ions can undergo a displacement reaction with copper ions, thereby completing the adsorption process of copper ions. In the process of adsorption of copper ions by hydrogel beads, it was found that the maximum adsorption capacity of hydrogel beads for copper ions in wastewater was 47.50 mg/g; the adsorption of copper ions was a spontaneous thermal adsorption process; and the adsorption mechanism included physical adsorption, ion exchange, surface complexation, electrostatic attraction, and precipitation [52]. When CS-based hydrogel beads are utilized to adsorb other metal ions in wastewater, the adsorption mechanism is analogous to that of copper ions. For instance, modified CS was used to prepare hydrogel beads capable of adsorbing lead ions. The results demonstrate that the prepared hydrogel beads not only adsorb lead ions efficiently but can also be reused at least ten times, making them an efficient and environmentally friendly option [53]. From the point of view of adsorption efficiency, it can be seen that, after repeated use of hydrogel beads 10 times, the adsorption efficiency of lead ions only decreased from the initial 259.68 mg/g to 217.38 mg/g. By analyzing the adsorption isotherm, it was found that the Langmuir isotherm model of the adsorption data of lead ions by hydrogel beads was in good agreement, and the adsorption process followed the pseudo-second-order kinetic model. Additionally, some researchers have employed CS-based hydrogel beads to adsorb certain charged ionic groups. For example, CS-based hydrogel beads have been used to adsorb phosphate groups in electroplating wastewater [54].

#### 3.3.2. CS-Based Hydrogel Beads Adsorbed Dyes and Drugs in Wastewater

Dyes in wastewater pose a significant environmental challenge, prompting researchers to frequently employ CS-based hydrogel beads for dye adsorption. For instance, CS has been utilized to prepare hydrogel beads capable of selectively absorbing anionic dyes, which exhibit both reusability and bacteriostatic properties [55]. It was proved that hydrogel beads have very fast absorption capacity and excellent adsorption properties for dyes by the experiments of adsorption kinetics such as pseudo-first- and pseudo-second-order kinetics. The adsorption process conforms to the pseudo-second-order model and the Langmuir isotherm model, which belongs to the homogeneous monolayer adsorption through the chemisorption process. Researchers have also investigated the preparation of hydrogel beads using the ionic gelation method, which enables the adjustment of CS content to enhance the beads’ affinity for either cationic or anionic dyes [56]. The experimental results show that the adsorption efficiency of hydrogel beads on dyes conforms to pseudo-second-order kinetics, and the adsorption of anions and cations involves chemisorption and multi-step adsorption. Beyond dye adsorption, CS-based hydrogel beads play a crucial role in the removal of pharmaceutical compounds from wastewater. Notably, some researchers have developed CS-based hydrogel beads incorporating iron hydroxide specifically for the adsorption of ibuprofen in wastewater [57]. Additionally, other studies have demonstrated the preparation of hydrogel beads combining humic acid and CS, which effectively adsorb three fluoroquinolones simultaneously, showing excellent adsorption performance [58]. The dense internal structure of CS-based hydrogel beads, coupled with the presence of various charged groups, provides numerous adsorption sites for both dyes and pharmaceutical compounds. Furthermore, the electrostatic interactions between CS-based hydrogel beads and dye molecules contribute to more efficient dye removal from wastewater. These findings highlight the substantial application potential of CS-based hydrogel beads in the adsorption of dyes and pharmaceutical compounds from wastewater.

In summary, CS-based hydrogel beads have demonstrated extensive applications and remarkable efficacy in food preservation, medical fields, and wastewater treatment. The porous structure of CS-based hydrogel beads provides abundant adsorption sites for various contaminants, including heavy metal ions, pharmaceutical compounds, and dyes in wastewater. Furthermore, the presence of charged groups within the hydrogel beads enables enhanced adsorption capabilities, particularly for anionic dyes, through improved molecular interactions. Future research directions may focus on the development of advanced chemical crosslinking agents for hydrogel bead preparation or the implementation of chemical modification techniques to increase the density of charged groups within the hydrogel matrix. These innovative approaches hold significant promise for optimizing the performance of CS-based hydrogel beads in environmental applications. Table 1 comprehensively summarizes the diverse applications of CS-based hydrogel beads.

## 4. Prospects and Challenges

CS-based hydrogel beads exhibit promising application prospects across multiple domains. Primarily, their exceptional biocompatibility and biodegradability make them ideal candidates for drug delivery systems, enabling the encapsulation of pharmaceutical compounds within the gel matrix for targeted transport and controlled release at specific sites. Furthermore, CS-based hydrogel beads serve as effective food additives, enhancing both the sensory qualities and textural properties of food products. Their remarkable water retention capacity and inherent antimicrobial characteristics significantly contribute to extending food shelf life. In environmental applications, these hydrogel beads demonstrate remarkable efficacy in adsorbing and removing various contaminants, including heavy metal ions and organic pollutants, thereby offering innovative solutions for water treatment technologies. Additionally, CS-based hydrogel beads have found substantial utility in diverse fields such as agriculture and cosmetics. Looking ahead, advancements in extraction technologies, coupled with progress in mechanical engineering, are anticipated to reduce raw material costs and facilitate the establishment of automated production lines. These developments are expected to significantly enhance the cost-effectiveness of CS-based hydrogel bead manufacturing.

However, CS-based hydrogel beads exhibit certain limitations that warrant attention. A primary concern is their relatively weak mechanical properties, which often fail to meet the requirements for high-strength applications. Consequently, significant improvements in mechanical strength are necessary to expand their applicability across a broader range of scenarios. Additionally, the high production costs associated with large-scale manufacturing of CS-based hydrogel beads present another substantial challenge that requires resolution. Future research directions may include the development of large-scale syringe pumps utilizing 3D printing technology or leveraging advancements in mechanical engineering to enhance the commercial viability of these hydrogel beads. While CS-based films have gained widespread adoption and demonstrate gradual degradation post-utilization, there remains a notable gap in research concerning the environmental impact and service life of CS-based hydrogel beads after use. This represents a crucial area for future investigation and development.

## 5. Conclusions

In this paper, different preparation methods and materials used in the preparation of CS-based hydrogel beads are reviewed. The latest applications of Cs-based hydrogel beads in food science, medicine, and environmental protection are systematically analyzed. CS itself is rich in amino and hydroxyl functional groups, so the prepared CS-based hydrogel beads can adsorb anionic dyes and cationic dyes. Moreover, due to the large number of adsorption sites inside the CS-based hydrogel beads, it has a good effect on the adsorption of heavy metal ions to treat water pollution. At the same time, CS-based hydrogel beads have a unique three-dimensional network structure, which also plays a good role in the encapsulation of bioactive substances for food preservation and as a drug transport system. However, despite the wide application of CS-based hydrogel beads, there are some disadvantages. The mechanical properties of CS-based hydrogel beads are poor, and large-scale production lines do not have resumes at present, which cannot be mass-produced. In addition, the pollution of CS-based hydrogel beads to the environment and whether they can be reused have not been deeply explored in the current research. In the future, with the further development of 3D printing technology, the research and development of large-scale injection pumps, and the further development of mechanical engineering technology, it is possible to achieve large-scale production of CS-based hydrogel beads. At the same time, with the emergence of more and more new materials, the continuous combination and use of CS, the mechanical properties of CS-based hydrogel beads, service life and environmental friendliness and other problems have a great possibility to be solved. These are also important directions for the future research and development of CS-based hydrogel beads.

## Figures and Tables

**Table 1:** Application of hydrogel beads in food.

Samples	Apply	Reference
Application in strawberry preservation	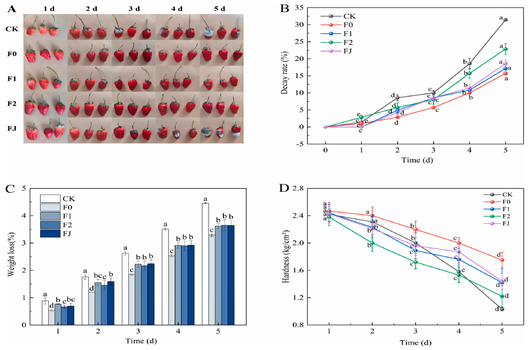	[41] Note: A represents the freshness of strawberries in different groups, and B–D represents the decay rate, weight loss and hardness of strawberries respectively
Hydrogel beads act as carriers of proanthocyanidins	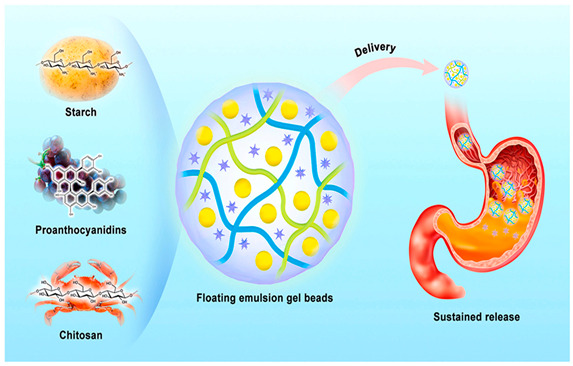	[11]
Hydrogel beads adsorb copper ions	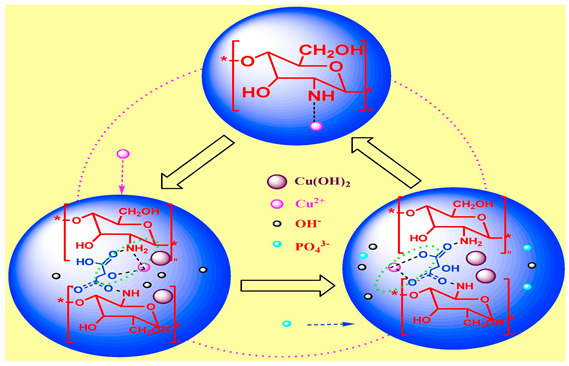	[59]

## Data Availability

Not applicable.
